# Photobiomodulation regulates astrocyte activity and ameliorates scopolamine-induced cognitive behavioral decline

**DOI:** 10.3389/fncel.2024.1448005

**Published:** 2024-09-20

**Authors:** Ji On Park, Namgue Hong, Min Young Lee, Jin-Chul Ahn

**Affiliations:** ^1^Department of Medical Laser, Graduate School of Medicine, Dankook University, Cheonan-si, Republic of Korea; ^2^Medical Laser Research Center, College of Medicine, Dankook University, Cheonan-si, Republic of Korea; ^3^Department of Otolaryngology-Head and Neck Surgery, College of Medicine, Dankook University, Cheonan-si, Republic of Korea; ^4^Department of Biomedical Science, College of Medicine, Dankook University, Cheonan-si, Republic of Korea

**Keywords:** scopolamine, Alzheimer’s disease, astrocyte, photobiomodulation, neuroinflammation

## Abstract

**Introduction:**

The pathophysiological mechanism of Alzheimer’s disease (AD) has not been clearly identified, and effective treatment methods have not yet been established. Scopolamine causes cholinergic dysfunction in the brain, including the accumulation of amyloid-beta plaques, thereby increasing oxidative stress and neuroinflammation, mimicking AD. Glial cells such as astrocytes have recently been identified as possible biomarkers for AD. Photobiomodulation (PBM) elicits a beneficial biological response in cells and tissues. PBM effects on the central nervous system (CNS) have been widely researched, including effects on astrocyte activity.

**Methods:**

In the present study, PBM was performed using light at the near-infrared wavelength of 825 nm. The Morris water maze and Y-maze tests were employed to evaluate cognitive function decline in a scopolamine-induced memory dysfunction model and its improvement with PBM. In addition, alteration of the mitogen-activated protein kinase (MAPK) pathway and immunofluorescence expression levels of active astrocytes were observed in the hippocampus, which is one of the areas affected by AD, to evaluate the mechanism of action of PBM.

**Results:**

A reduction in the neuronal cell death in the hippocampus caused by scopolamine was observed with PBM. Moreover, alteration of a MAPK pathway-related marker and changes in glial fibrillary acidic protein (an active astrocyte marker) expression were observed in the PBM-treated group. Finally, significant correlations between functional and histological results were found, validating the results.

**Discussion:**

These findings indicate the possibility of behavioral and histological improvement due to PBM in scopolamine-induced CNS alteration, which mimics AD. This improvement could be related to neuroinflammatory modulation and altered astrocyte activity.

## 1 Introduction

The incidence of neurodegenerative disorders tends to gradually increase with increasing life expectancy. Dementia, a common neurodegenerative disorder, is a disease associated with cognitive dysfunction of learning, memory, and problem-solving ability (Crawford and Higham, 2016), as well as accompanying psychotic symptoms such as anxiety, depression, impulsivity, and abnormal behavior. Alzheimer’s disease (AD) accounts for more than 75% of dementia cases (Hyman, 1997). In contrast to vascular dementia, which is caused by cerebrovascular disorders, the pathophysiological mechanism of AD has not been clearly identified, and effective treatment methods have not been established. AD causes extensive atrophy of the cortex and increased abundance of amyloid-beta plaques (Murphy and LeVine, 2010). In addition, neuroinflammation and reactive gliosis are associated with AD (Perry et al., 2010).

In this study, scopolamine hydrobromide was used to model AD (Pitsikas, 2015). Scopolamine is a drug that acts as an antagonist of the muscarinic receptor, a type of acetylcholine receptor. Acetylcholine is a major neurotransmitter in the nervous system that plays an important role in cognitive functions such as learning and memory. An increase in acetylcholinesterase (AChE), which breaks down acetylcholine, causes cholinergic dysfunction through the breakdown of acetylcholine into choline and acetyl CoA. In addition, cholinergic dysfunction is expected to cause cognitive dysfunction (Ghoneim and Mewaldt, 1977). Thus, scopolamine causes cholinergic dysfunction in the brain, leading to accumulation of amyloid-beta plaques, and thus facilitating increases in oxidative stress and neuroinflammation (Tang, 2019).

Astrocytes are a prevalent type of glial cell in the central nervous system (CNS). Astrocytes have diverse functions within the CNS, including contributions to synaptogenesis, neurotransmitter buffering, and inter- and intracellular communication (Nedergaard and Verkhratsky, 2012). Generalized astrogliosis, including hypertrophy, was observed in tissues of post-mortem AD patients (Oberheim et al., 2008). Recently, biomarkers related to diverse astrocyte activities have been reported. In addition, novel markers such as glial fibrillary acidic protein (GFAP) in the blood or CNS have been proposed for the clinical management of AD, and single-cell RNA maps of astrocyte-related genes have been introduced in AD animal models (Carter et al., 2019; Endo et al., 2022).

Photobiomodulation (PBM) elicits beneficial biological responses in cells and tissues (Waypa et al., 2016). PBM is used as a treatment for various diseases and disorders, and research results indicate that it is effective against brain diseases (Hamblin, 2016). For deep penetration of light energy, the use of red and near infrared light radiation is necessary (Mester et al., 1976). PBM can treat brain diseases by reducing neuroinflammation in the brain and reducing oxidative stress (DeTaboada et al., 2006). In our prior research, PBM has exhibited efficient modulation of synaptogenesis and neurogenesis in the CNS (Hong et al., 2022; Hong et al., 2023). In addition, astrocyte-specific modulation has been observed (Yoon et al., 2023; Yoon et al., 2021).

In the present study, PBM was performed with a light at the near-infrared wavelength of 825 nm. The Morris water maze (MWM) and Y-maze tests were employed to evaluate the decline of cognitive function in a model of scopolamine-induced memory dysfunction and its improvement with PBM. In addition, alterations to the mitogen-activated protein kinase (MAPK) pathway and immunofluorescence expression level of astrocytes were observed in the hippocampus, one of the areas affected by AD (Balestrieri et al., 2020; Fellgiebel and Yakushev, 2011; Luppi et al., 2022; Rao et al., 2022), which may elucidate the mechanism of action of PBM.

## 2 Materials and methods

### 2.1 Animal and housing

Seven-weeks old male C57BL/6 mice were purchased from Narabiotek (Seoul, South Korea). The mice were given a one-week acclimation period to adjust to their new environment. Following this, they underwent training for animal behavior tests for two weeks. A total of 20 mice, 5 per group, were used in the experiment. All *in vivo* experimental procedures were certified and approved by the Institutional Animal Care and Use Committee (IACUC) of Dankook University. All mice were housed under a controlled environment with 12-h light/dark cycles and temperature.

### 2.2 Scopolamine-induced cognitive dysfunction animal model

Scopolamine hydrobromide was purchased from Sigma-Aldrich (St. Louis, MO, USA). Ten-week-old male C57BL/6 mice were used for the animal model of cognitive dysfunction. The animals were housed in a temperature- and light-controlled room (12/12-h dark/light cycle). In this study, the mice were divided into four groups: a group receiving saline treatment (Control); low-level laser therapy-treated group (Laser); scopolamine-treated group (Sco.); and scopolamine- and laser-treated group (Sco. + Laser). The solution of scopolamine hydrobromide was prepared using saline. Solutions were prepared 3 times per week and injected intraperitoneally (i.p., 2 mg/kg). Mice were administered scopolamine for 12 weeks. Vehicle groups were injected with an equal volume of saline.

### 2.3 Low-level laser therapy treatment

**FIGURE 1:**
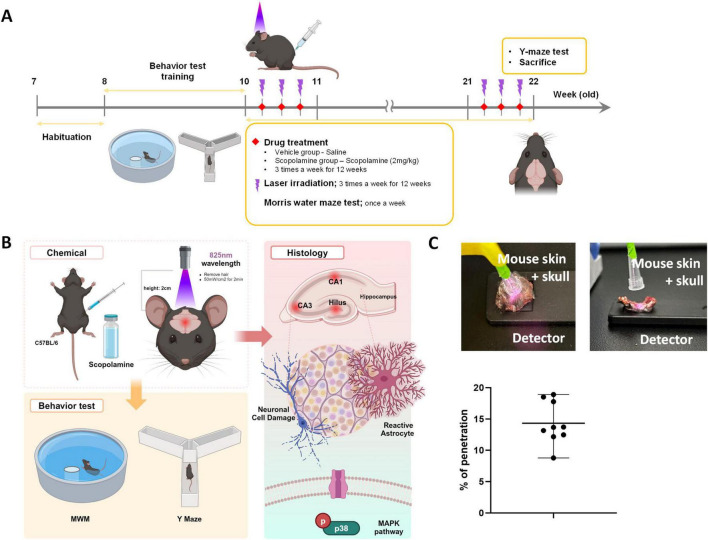
Diagram of the experimental process. **(A)** After a 1-week adaptation period, C57BL/6 mice were treated with vehicle (i.p., saline) or scopolamine (i.p., 2 mg/kg) with or without laser (central wavelength: 825 nm, 70 mW, 2 min) 3 times per week for 12 weeks. **(B)** Schematic diagram of laser irradiation. We removed hair from the heads of mice and applied the laser to the skin of the head. **(C)** Laser penetration rate at the wavelength of 825 nm.

In this study, we used a laser with a central wavelength of 825 nm (Wontech, Daejeon, South Korea) for the scopolamine-induced cognitive dysfunction model. PBM using low-level laser therapy (LLLT) was administered to mice 3 times per week for 12 weeks. LLLT was administered 30 min after scopolamine treatment. Distance to the head from the end of a laser fiber was 5 cm, and 70 mW/cm^2^ power density was applied for 2 min. The energy at the mouse head was measured using a laser power meter (PD-300 and VEGA power meter, Ophir). Laser penetration rate is shown in Figure 1. To measure laser penetrance, we sampled the mouse scalp and skull, placed a laser power detector underneath, and measured the power of the light after it passed through the mouse scalp and skull. The penetrance was calculated by determining the reduction in energy from the end of the optical fiber.

### 2.4 Cognitive function testing

#### 2.4.1 Morris water maze

The water maze apparatus consisted of a circular pool 120 cm in diameter, filled with water at 23 ± 2°C to cover a platform. The platform was located in the center of the northeast quadrant. Each mouse’s trial was monitored with a video camera connected to a computer. Mice were trained twice daily for 2 weeks. During each training trial, mice that did not find the platform within 60 s were placed on the platform for 30 s at the end of the trial. The test trial was performed 3 weeks after scopolamine and LLLT administration. During each test trial, the time required to escape onto the hidden platform was recorded. Mice were allowed to locate the hidden platform for 180 sec. EthoVision XT 15 software (Noldus Information Technology, Wageningen, the Netherlands) was used for analysis of behavioral parameters.

#### 2.4.2 Y-maze test

The Y-maze test was conducted using a three-arm maze with angles of 120° between arms; the arms were 35 cm in length and 3 cm in width, with walls that were 15 cm high. The maze floor and walls were constructed of dark opaque polyvinyl chloride plastic. At 30 min after scopolamine and PBM treatment, mice were placed at the end of one arm and allowed to move freely through the maze for 5 min. Alternation was defined as successive entries into three arms on overlapping triplet sets, i.e., patterns of ABC, BCA, and CAB were recorded as alternations, but not ACA. Spontaneous alternation behavior reflects spatial working memory, which is a form of short-term memory (1). Spontaneous alternation (%) was defined as the ratio of the number of alternations to the number of total arm entries minus 2, as expressed in the following equation:


Spontaneousalternation(%)=

$$\,\frac{T\,o\,t\,a\,l\,N\,u\,m\,b\,e\,r\,o\,f\,a\,l\,t\,e\,r\,n\,a\,t\,i\,o\,n\,s}{T\,o\,t\,a\,l\,a\,r\,m\,e\,n\,t\,r\,i\,e\,s-2}\times 100$$

EthoVision XT 15 software (Noldus Information Technology, Wageningen, the Netherlands) was used for analysis of behavioral parameters.

### 2.5 Tissue staining

#### 2.5.1 Tissue processing

Mouse brains were removed and perfused with 4% paraformaldehyde. The brains were rinsed and cryoprotected using a series of 10, 20, and 30% sucrose in phosphate buffered saline (PBS) at 4°C for 2 days. Brain tissues were frozen in Tissue-Tek solution and stored at −80°C until sectioning. Tissues were sectioned with a Cryostat Microtome CM1850 (Leica Biosystems, Wetzlar, Germany) into 20-μm thick coronal sections.

#### 2.5.2 Cresyl violet staining

Brain sections were mounted on slides and treated in a series of solutions for cresyl violet (CV) staining. Slides were rehydrated in a graded ethanol series (100, 95, 90, and 80% ethanol). After rehydration, slides were stained with a pre-warmed 0.3% CV solution for 20 min at room temperature. After decolorization with a solution of 95% ethanol and 0.3% glacial acetic acid, the slides were dehydrated using 100% ethanol, followed by 100% xylene. Finally, the slides were covered with glass coverslips using Permount mounting medium. The number of Cresyl violet-positive healthy neurons was quantified by manually counting the neurons using at least three staining images per animal with a 200× lens.

#### 2.5.3 Immunofluorescence

Free-floating brain sections were sufficiently washed with PBS, permeabilized with 0.5% Triton X-100 in PBS, and then blocked with 5% bovine serum albumin in PBS containing 0.4% Triton X-100. Brain section tissues were incubated overnight at 4°C with antibodies. Anti- phospho-p38 (1:250; #9215, RRID:AB_331762, Cell Signaling Technology, Massachusetts, United States), anti-phospho-p44/42 MAPK (1:250; #9101, RRID:AB_331646, Cell Signaling Technology, Danvers, MA), anti-neuronal nuclear protein (NeuN) (1:500; MAB377,RRID:AB_2298772, EMD Millipore) and anti-GFAP (1:500; MAB360, RRID:AB_11212597, Sigma-Aldrich, St. Louis, MO, USA) were used in the present study. After washing in PBS, brain sections were incubated with Alexa Fluor 488 goat anti-mouse IgG (1:500; Invitrogen, Carlsbad, CA, USA) and Alexa Fluor 555 goat anti-mouse IgG (1:500; Invitrogen, Carlsbad, CA, USA) for 2 h at room temperature. Slides were covered with glass coverslips using Vectashield Mounting Medium (Vector Laboratories, Inc., Burlingame, CA, USA). Stained slides were imaged using a confocal microscope (Olympus Corporation, Tokyo, Japan). Quantification was performed using ImageJ software version 1.52 (National Institutes of Health, USA). All fluorescence images were quantified by using ImageJ software (National Institutes of Health, USA, version 1.52) to separate each color channel, followed by measuring the average fluorescence intensity of each fluorescence channel image.

### 2.6 Primary neuronal culture and Real-time polymerase chain reaction (RT-PCR)

For the RT-PCR analysis, primary cells obtained from rat pup were used (Supplementary Figure 1A). Rat hippocampal and cortical neurons were grown in primary culture as described previously (Hong et al., 2023). Briefly, fetuses were removed from maternal rats anesthetized with 16.5% urethane on embryonic day 17. Hippocampi were dissected and placed in Ca2+- and Mg2+-free HEPES-buffered Hanks’ salt solution (HHSS, pH 7.45, 20 mM HEPES, 137 mM NaCl, 1.3 mM CaCl2, 0.4 mM MgSO4, 0.5 mM MgCl2, 5.0 mM KCl, 0.4 mM KH2PO4, 0.6 mM Na2HPO4, 3.0 mM NaHCO3, and 5.6 mM glucose). Cells were dissociated by trituration using a 5-ml pipette and a flame-narrowed Pasteur pipette. Cells were then, pelleted and resuspended in neurobasal medium without L-glutamine with 2% B27 supplement, 0.25% Glutamax I and penicillin/streptomycin/amphotericin B (100 U/ml, 100 and 0.025 μg/mL, respectively). Dissociated cells were then plated onto 6 well plate at a density of 200,000 cells/well. The 6 well plate was pre-coated with Poly-D-lysine (0.5 mg/ml; Sigma-Aldrich, St. Louis, MO, USA). Neurons were grown in a humidified atmosphere at 37°C with 10% CO2 and 90% air, at pH 7.4. They were fed on days 3, 7, and 10 by replacing 75% of spent media with fresh media. Experimental schedule is shown in Supplementary Figure 1B. Representative image of co-culture of two different cells, astrocyte and neuron are shown in Supplementary Figure 1C. Total RNA was extracted using RiboEX (GeneAll, Seoul, Republic of Korea). Before cDNA synthesis, the RNA concentrations were measured using a NanoDrop spectrophotometer (ND-1000; NanoDrop, Wilmington, DE, USA); 1 μg of total RNA was reverse-transcribed using Hyperscript™ 2X RT Master mix (GeneAll). qRT-PCR was performed using AccuPower^®^ 2 GreenStar™ qPCR Master Mix (Bioneer, Daejeon, Republic of Korea) and gene-specific primers (Supplementary Table 1) in an RT-PCR system (ABI 7500; Applied Biosystems, Foster City, CA, USA). Each target gene expression level was normalized to endogenous GAPDH using the formula: [ΔCt = Ct (target gene)—Ct (GAPDH)]. The 2^–ΔΔCt^ method was applied to calculate the relative quantification value of target genes to control samples. Primer sequences are shown in Supplementary Table 1.

### 2.7 Statistical analysis

Data are expressed as the group mean ± standard error of the mean (SEM). Prism 7 (GraphPad Software, San Diego, CA, USA) was used for one-way analysis of variance with Bonferroni’s post-hoc test. Before conducting the ANOVA test, we performed normal (Gaussian) distribution tests, including the D’Agostino-Pearson omnibus normality test, Anderson–Darling test, Shapiro–Wilk normality test, and Kolmogorov-Smirnov normality test. The ANOVA test was conducted on graphs where normal distribution was confirmed. The Pearson correlation test was used for assessment of relationships between function and histology. Differences between groups were considered significant at *p* < 0.05. Significance levels: *p ≤ 0.05, **p ≤ 0.01, ***p ≤ 0.001, ****p ≤ 0.0001.

## 3 Results

### 3.1 Decline in cognitive function after scopolamine application and its reversal with PBM

**FIGURE 2:**
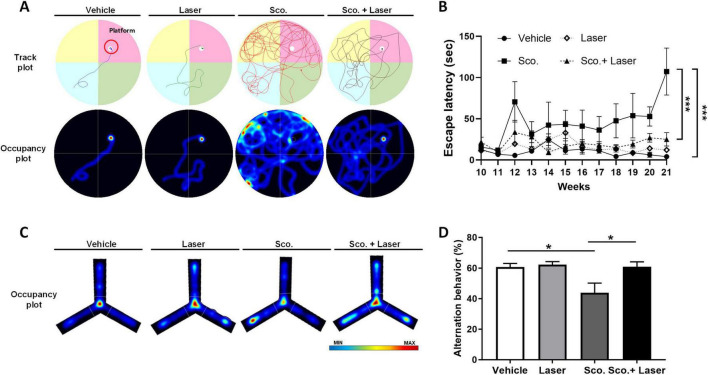
Effects of photobiomodulation (PBM) on scopolamine-induced cognitive dysfunction and behavioral variations in the experimental groups. To evaluate spatial working memory, the Morris water maze (MWM) and Y-maze tasks were used. Performance of mice on the MWM and Y-maze tests. **(A)** Representative track plots and occupancy plots from a MWM test trial. The track plots and occupancy plots indicate the path covered by the mice during the test. **(B)** Group latency times to find the platform in the MWM test. Graph showing latency to reach the hidden platform during test trials. Data are presented as mean ± SEM. Significance: *p < 0.05 **(C)** Representative occupancy plots from the Y-maze test. Red color represents increased time spent and blue color represents minimal time spent in an area during the trial. **(D)** Spontaneous alternation behaviors in the Y-maze test trial. Data are presented as mean ± SEM. Significance: *p < 0.05, ***p ≤ 0.001. LLLT, low-level laser therapy = PBM.

To evaluate the effects of PBM on cognitive function in scopolamine-treated mice, we performed the MWM and Y-maze tests. MWM tests were performed to confirm spatial memory function and Y-maze tests were performed to confirm short-term memory function. Before the MWM test was performed, mice were trained to learn the location of the platform. Mice were trained twice daily for 1 week to remember the location of the platform. The MWM test was performed once weekly for 3 weeks after scopolamine administration. The Y-maze test was performed once before sacrifice. During the behavioral tests, all track plots that mice moved along were recorded with a camera and software (Figure 1). In the MWM test, the scopolamine-treated group showed increased escape latency, taking longer to find the hidden platform compared to the vehicle group. However, PBM treatment after scopolamine administration significantly reduced the mean escape latency time associated with scopolamine (Figures 2A, B). In the Y-maze test, short-term memory function was evaluated based on the percent of correct spontaneous alternations (SAP). Scopolamine decreased SAP compared to the vehicle group. PBM treatment after scopolamine administration (2 mg/kg) significantly increased the amount of correct SAP (Figures 2C, D). These results indicate that PBM treatment of scopolamine-treated mice restored cognitive function, including both spatial memory and short-term memory. To confirm this result histologically, the hippocampus of each animal was observed.

### 3.2 Reduction in neuronal cell population after scopolamine application and its regulation by PBM

**FIGURE 3:**
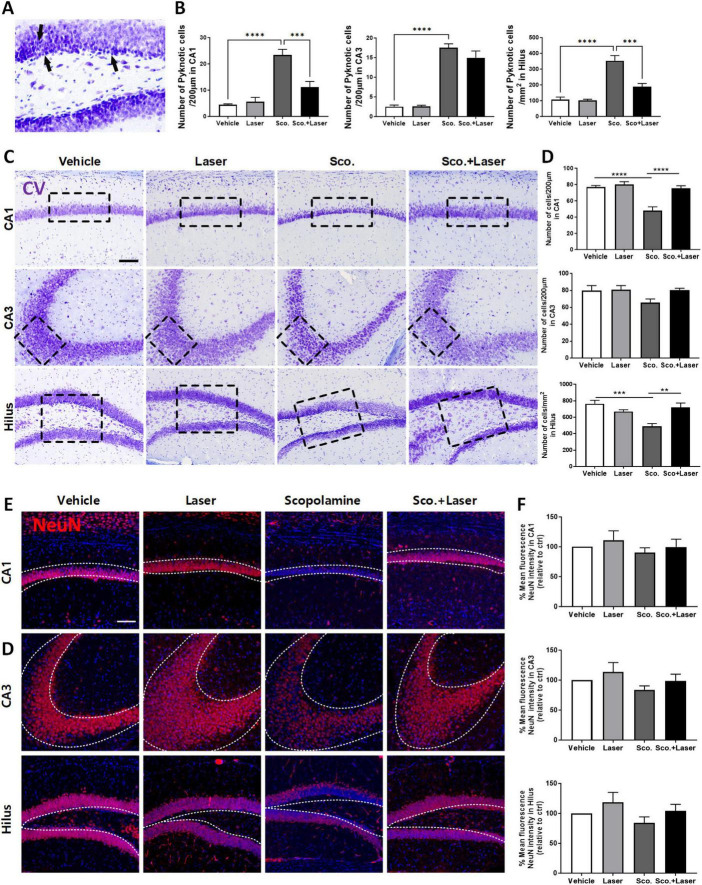
Neuroprotective effect of photobiomodulation (PBM) in hippocampal regions of the scopolamine-induced cognitive dysfunction model evaluated through cresyl violet staining. Images and quantitative analysis of cresyl violet (CV)-stained hippocampal neurons in the experimental groups. Neuronal morphology and distribution were observed in the CA1 and CA3 regions and hilus after CV staining. **(A)** Representative image of pyknotic cell. The scopolamine treated group, being darker stained with shrunk and tightly nuclei and cell bodies, indicative of pyknotic morphology. **(B)** Quantitative analysis of pyknotic cell in each group. Data are presented as mean ± SEM. Significance: ***p ≤ 0.001, ****p ≤ 0.0001. CA1 ANOVA; *F*(3, 28) = 22.80, *p* < 0.0001, CA3 ANOVA; *F*(3, 19) = 57.96, *p* < 0.0001, Hilus ANOVA; *F*(3, 28) = 24.19, *p* < 0.0001. **(C)** Representative images of CV-stained sections. PBM administration 30 min after scopolamine treatment protected hippocampal neuronal cells. Scale bar: 100 μm **(D)** Quantitative analysis of normal neuronal cells in each group. The target areas are the CA1, CA3 and DG regions. Data are presented as mean ± SEM. Significance: **p ≤ 0.01, ***p ≤ 0.001, ****p ≤ 0.0001. CA1 ANOVA; *F*(3, 28) = 18.55, *p* < 0.0001, CA3 ANOVA; *F*(3, 19) = 2.175, *p* = 0.1244, Hilus ANOVA; *F*(3, 28) = 8.415, *p* = 0.0004. **(E)** Expression pattern of NeuN in target areas. **(F)** Quantitative analysis of mature neuron in each group. LLLT, low-level laser therapy = PBM.

CV staining was performed for histopathological analysis to assess the degree of neuronal cell death. Pyknotic cells exhibit irreversible condensation of chromatin in the nucleus, leading to cell apoptosis. Pyknotic cells were observed in the hilus, CA1 and CA3 hippocampal subfields of scopolamine-treated mice (Figures 3A, B). In the scopolamine-treated group, CV-positive cells were detected in the hilus at 488.46 cells/200 μm, CA3 at 65.57 cells/200 μm and CA1 at 48 cells/200 μm. Compared to the vehicle group, CV-positive cell abundance levels were significantly reduced in the hilus and CA1 subregions. This result is supported by a similar expression pattern of NeuN in these areas (Figure 3C). The results indicate that scopolamine promotes neuronal loss in the hilus, CA1 and CA3 regions. However, PBM treatment preserved neuron survival and prevented scopolamine-induced neuronal loss in those hippocampal regions of scopolamine-induced cognitive dysfunction model animals. In the PBM treatment group, CV-positive cells were detected in the hilus at 719 cells/200 μm, CA3 at 80.55 cells/200 μm and CA1 at 75.37 cells/200 μm (Figures 3C, D). The abundance of CV-positive cells significantly increased in the hilus and CA1 region of the PBM-treated group compared to the scopolamine-treated group. The study subsequently investigated the influence of PBM on neuronal apoptosis by examining changes in mRNA levels related to representative apoptosis markers. The relative mRNA expression of the apoptotic marker Bax showed a tendency to increase in the scopolamine-treated group, while it tended to decrease in the PBM-treated group (Supplementary Figure 1D). The expression level of the anti-apoptotic marker Bcl2 increased in the scopolamine-treated group compared to the vehicle group, and it showed a slight increase in the PBM-treated group (Supplementary Figures 1E, F). This result is supported by a similar expression pattern of the alive mature neuronal marker NeuN in these areas (Figures 3E, F). The results indicate that scopolamine promotes neuronal loss in the hilus, CA1 and CA3 regions.

### 3.3 Alteration of the MAPK pathway and increase in the activated astrocyte population in scopolamine-treated animals and their regulation by PBM

**FIGURE 4:**
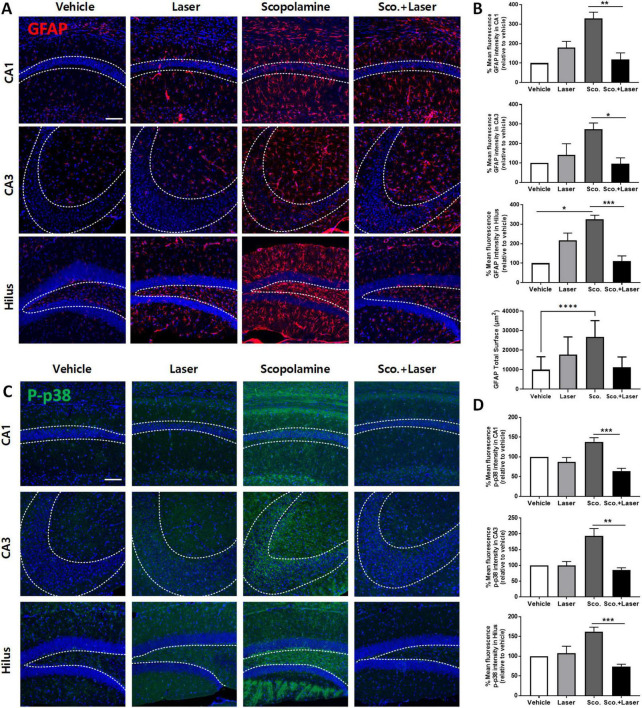
Effect of photobiomodulation (PBM) on astrocyte activation and level of phosphorylated p38 expression in a scopolamine-induced cognitive dysfunction model. Images and quantitative analysis of anti-GFAP immunofluorescence in the experimental groups. **(A)** Representative images of anti-GFAP-stained brain sections. Scale bar; 100 μm. **(B)** Scopolamine treatment increased anti-GFAP (red) in hippocampal regions, as evidenced by immunofluorescence staining. Graph showing the mean intensity of GFAP. Also the GFAP total surface bar graph represents the measured area of GFAP-positive cells expressed in all hippocampus sub-regions. Data are presented as mean ± SEM. Significance: *p < 0.05, **p ≤ 0.01, ***p ≤ 0.001, ****p ≤ 0.0001. CA1 ANOVA; *F*(3, 12) = 8.782, *p* = 0.0024, CA3 ANOVA; *F*(3, 12) = 4.540, *p* = 0.0239, Hilus ANOVA; *F*(3, 12) = 12.5, *p* = 0.0005, GFAP total surface ANOVA, *F*(3, 68) = 22.24, *p* < 0.0001. **(C)** Representative confocal microscopic images of the immunoreactivity of p-p38 (green) in scopolamine-treated mouse brains. Scale bar; 100 μm. **(D)** Analysis of the fluorescence intensity of p-p38 in the CA1, CA3 and hilus regions. Data are presented as mean ± SEM. Significance: *p < 0.05, **p ≤ 0.01, scale bar; 100 μm Graph showing the mean intensity of GFAP. Data are presented as mean ± SEM. Significance: *p < 0.05, **p ≤ 0.01, ***p ≤ 0.001. CA1 ANOVA; *F*(3, 12) = 11.02, *p* = 0.0009, CA3 ANOVA; *F*(3, 12) = 7.821, *p* = 0.0037 Hilus ANOVA; *F*(3, 12) = 10.84, *p* = 0.001, scale bar; 100 μm, LLLT: low-level laser therapy = PBM.

Glial cells (astrocytes and microglia) play an important role in inflammation. When nerve cells are damaged, they transform into reactive forms. Reactive astrocytes weaken synaptic support and release inflammatory and neurotoxic factors, inducing neuronal death and brain atrophy, thus causing severe dementia. Reducing the abundance of reactive glial cells is important for normal neuronal cell function. Therefore, we evaluated the effects of PBM on astrocytic changes by testing anti-GFAP immunoreactivity, an astrocyte marker. After scopolamine treatment, more GFAP-labeled reactive astrocytes were observed in the hilus, CA3, and CA1 regions. These results indicate that astrocytes were affected by scopolamine-induced brain changes and switched to their reactive form. In the PBM-treated group after scopolamine administration, the number of anti-GFAP-labeled reactive astrocytes was significantly decreased in the hilus, CA3, and CA1 regions compared to the scopolamine-treated group, with no significant difference in GFAP immunoreactivity between the vehicle- and PBM-treated groups (Figures 4A, B). The difference of IBA1 positive cell characteristics among groups showed similar patterns (Supplementary Figure 2). The role of phosphorylated (p)-p38 MAPK in neurodegenerative disorders is critical, as this factor triggers microglial activation, neuroinflammation, oxidative stress, and apoptosis. We evaluated the level of activated p-p38 MAPK in hippocampus subfields using immunofluorescence. The mean fluorescence intensity in the region indicated by the dotted line was measured and compared for each group. Phosphorylation of p38, as analyzed through immunofluorescence, was elevated in the hilus, CA3, and CA1 regions in the scopolamine treatment group. PBM treatment after scopolamine administration reduced p-p38 mean fluorescence intensity (Figures 4C, D) In the scopolamine treatment group, p-JNK fluorescence intensity was higher compared to the vehicle group. On PBM treatment after the scopolamine administration group, the fluorescence intensity of p-JNK decreased (Supplementary Figure 3). However, along with the p-ERK which showed no difference among groups, p-JNK difference among groups were not statistically significant.

### 3.4 Plot analysis and correlations between cognitive behavioral test results and histology

**FIGURE 5:**
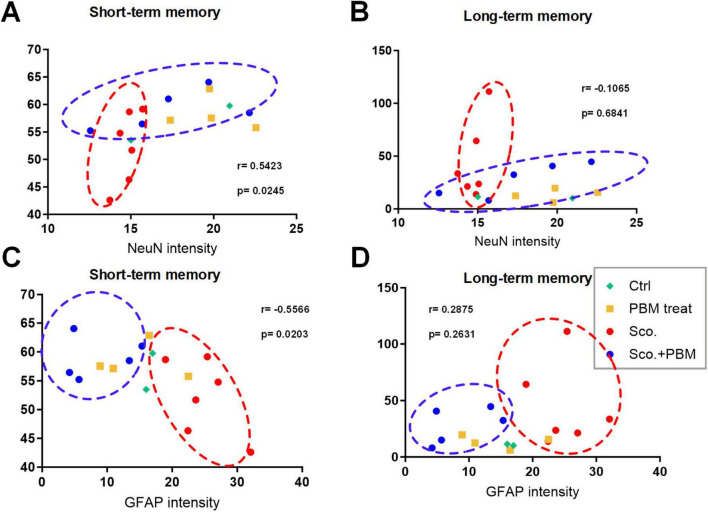
Plots of functional and histological tests for model animals. Animals are plotted based on the observed epifluorescence intensities of NeuN and GFAP in hippocampus regions and memory function evaluation results from the MWM test (lower = better performance) and Y-maze test (higher = better performances). **(A)** Plot of animals based on Y-maze test outcomes and NeuN intensity. **(B)** Plot of animals based on MWM test outcomes and NeuN intensity. **(C)** Plot of animals based on Y-maze test outcomes and GFAP intensity. **(D)** Plot of animals based on MWM test outcomes and GFAP intensity.

We performed two behavioral tests, MWM and Y-maze, to assess long- and short-term memory functions, respectively. Behavioral and histological test outcomes for each animal were plotted (Figure 5). On plots of MWM and histological results, the scopolamine group (red dotted circle) were located in areas of higher MWM, higher GFAP intensity, and lower NeuN. Meanwhile, the scopolamine and laser group (blue dotted circle) were plotted in areas of lower MWM, lower GFAP intensity, and higher NeuN. On Y-maze test and histological result plots, the scopolamine group (red dotted circle) was associated with lower Y-maze score, higher GFAP intensity, and lower NeuN. Meanwhile, the scopolamine and laser group (blue dotted circle) fell into areas of higher Y-maze score, lower GFAP intensity, and higher NeuN. These results correspond to the results outlined above demonstrating alteration of outcomes with laser intervention.

Correlation analysis was performed between behavioral tests and mature neuron and glial cell intensities, NeuN (a marker of mature neurons) and GFAP (a marker of reactive astrocytes). The correlation between Y-maze test results and NeuN intensity was significant and positive (*r* = 0.5423, *p* = 0.0245, 95% confidence interval [95%CI] = 0.08334 to 0.8114). The correlation between Y-maze test and GFAP intensity was shown as a negative correlation (*r* = −0.5566, *p* = 0.0203, 95%CI = −0.8183 to −0.1036). However, no significant correlations were found between MWM test results and NeuN or GFAP intensity (*r* = −0.1065, *p* = 0.6841, *r* = 0.2875, *p* = 0.2631). The lack of correlations between MWM results and histology could be due to small variance among most animals, with the exception of several animals in the scopolamine group. In this study, we analyzed the correlation between long/short-term memory and mature neuron and glial cell. Pearson correlation coefficient (Pearson’s *r*) is correlation coefficient that measures linear correlation between two set of data. The Pearson’s *r* always has value between –1 and 1. As value closer to 1, the data set shown positive correlation and the closer to –1 shown negative correlation. In this study, the *r* value between NeuN intensity and short-term memory shown *r* = 0.5423, shown positive correlation. And the GFAP intensity and short-term memory shown *r* = −0.5566, shown negative correlation. These results suggest that there is a highly correlation between short-term memory function and mature neurons and astrocytes.

## 4 Discussion

In the present study, we performed PBM treatment on the scopolamine-induced memory dysfunction animal model, to improve cognitive function and neuronal viability. Our results demonstrated that chronic administration (12 weeks) of scopolamine significantly damaged cognitive function including spatial memory, short- and long-term memory functions, and neuronal cell viability. However, chronic treatment with PBM after scopolamine administration significantly improved cognitive function and cell viability, while reducing reactive astrocytes.

In the MWM test, the escape time was significantly shorter for the PBM treatment group than the scopolamine treatment group. These results indicate that PBM improved long-term memory function that had been damaged by scopolamine administration. In the Y-maze test evaluating short-term spatial memory, SAP behavior increased in the scopolamine administration with PBM treatment group compared to the scopolamine-only group. These behavioral results demonstrate that PBM treatment of a memory dysfunction animal model improved short- and long-term spatial memory. Alteration of MAPK pathway factors is observed in AD patients (Kim and Choi, 2010). Studies have shown that the phosphorylated form of the MAPK factor p38 is present at increased levels in neurons of AD patients and AD animal models compared to healthy tissues (Zhu et al., 2002). In this study, activity of MAPK p-p38 tended to increase in various hippocampus regions of the scopolamine-induced memory dysfunction model, while its activity decreased in the PBM-treated group. Phosphorylation (activation) of p38 MAPK leads to the induction of cytokine production in glial cells (Raingeaud et al., 1995) and induces accumulation of GFAP in astrocytes (Zhuang et al., 2006). Reactive astrocytes further reduce synaptic support and may exacerbate microglial activation (Steele and Robinson, 2012). Reactive glia release inflammatory and neurotoxic factors, which induce neuronal death and brain atrophy, thereby causing severe dementia (Kempuraj et al., 2016).

Scopolamine is a muscarinic receptor antagonist widely used to study cognitive deficits in animal models (Klinkenberg and Blokland, 2010; Flood and Cherkin, 1986). Blockade of the muscarinic receptor may induce cholinergic disorder, which in turn causes a pattern of cognitive dysfunction in AD patients. The cholinergic system modulates hippocampal plasticity for both neurons and glial cells including astrocytes and microglia. Astrocytes are responsible for the maintenance and support of neurons. Astrocytes can also regulate immune responses in the CNS by detecting danger signals and secreting cytokines and chemokines. In a neuroinflammatory state, astrocytes respond with migration, hypertrophy, and increased GFAP levels. PBM can decrease the number of anti-GFAP-positive astrocytes. Previous studies have shown that astrocytes respond to acetylcholine and affect short-term and long-term cognitive function through alteration of synaptic function and plasticity (Hong et al., 2022; Hong et al., 2023; Yoon et al., 2023; Yoon et al., 2021). Therefore, reducing the reactivity of astrocytes may aid in recovery from cholinergic disorders and memory deficits. A1 and A2 astrocytes are two types of reactive astrocytes. The reactive astrocytes are induced by injury, neuroinflammation, and neurodegenerative disease. Reactive astrocytes can switch to either the pro-inflammatory, neurotoxic A1 astrocytes or the anti-inflammatory, neuroprotective A2 astrocytes (Nedergaard and Verkhratsky, 2012). The A1 astrocyte produces pro-inflammatory molecules and neurotoxins and the A2 astrocyte provides neurotrophic support and modulates inflammatory responses. A2 astrocytes upregulate anti-inflammatory and neurotrophic factors (Liddelow and Barres, 2017). A1 astrocytes are neurotoxic and identified in the brains of AD patients {Santiago-Balmaseda, 2024 #82}. In this study, we did not distinguish between the two types of astrocytes but only studied the tendency of total reactive astrocytes. In a further study, need to study whether PBM affects A1 and A2 astrocytes in a scopolamine-induced cognitive dysfunction animal model. Various previous studies have focused on the relationship between cognitive impairment and neuroinflammation induced by reactive gliosis. In this study, we focused on the relationship between cognitive function, neurons, and reactive astrocytes. However, there have also been reports linking neuroinflammation induced by M1 reactive microglia to neuronal damage (AmeliMojarad and AmeliMojarad, 2024). Therefore, it is necessary to elucidate the mechanisms underlying neuroinflammation induced not only by reactive astrocytes but also by reactive microglia in relation to neuronal damage in future studies. In summary, PBM treatment showed a therapeutic effect on scopolamine-induced memory dysfunction in an animal model. After PBM treatment, cognitive function and spatial memory function were improved. Moreover, PBM improved neuronal cell viability and reduced neuroinflammation.

In conclusion, in the scopolamine-induced memory dysfunction model, the group treated with PBM showed significant improvement in cognitive function. PBM treatment apparently controlled the activation level of neuroinflammation factors and activation of astrocytes, thereby improving cognitive function. Currently, no effective cure is available for neurodegenerative diseases such as AD. Drugs including donepezil, tacrine, and rivastigmine have been developed for the treatment of AD (Saxena et al., 2008), but these drugs exhibit side effects such as toxicity, vomiting, and nausea in humans. Therefore, a treatment for dementia with less toxicity and side effects is needed (Francis et al., 1999; Rountree et al., 2009). PBM, a non-invasive treatment method, may be an effective treatment for cognitive dysfunction diseases including AD.

## Data Availability

The data supporting the findings of this study are available from the corresponding author upon reasonable request.

## Supplementary material

Supplementary Figure 1 Analysis of the relative mRNA expression of Bax, Bcl2, and Caspase-3 in a scopolamine-induced cognitive dysfunction model. **(A)** Illustration of harvesting two different primary cells (astrocyte, neuron). **(B)** Experimental schedule of co-culture study. **(C)** Representative image of co-culture cells (astrocyte, neuron). Scale bar; 100 μm. **(D)** The quantitative analysis of the relative level of Bax. **(E)** The quantitative analysis of the relative level of Bcl2. **(F)** The quantitative analysis of the relative level of Caspase-3.

Supplementary Figure 2 The epifluorescence analysis of microglial cells (IBA1). Images and quantitative analysis of anti-IBA1 immunofluorescence in the experimental groups. **(A)** Representative images of anti-IBA1-stained brain sections. Scale bar; 50 μm. **(B)** Scopolamine treatment increased anti-IBA1 (Green) in hippocampal regions, as evidenced by immunofluorescence staining. Graph showing the number of IBA1-positive cells. Also, the IBA1 total surface bar graph represents the measured area of IBA1-positive cells expressed in all hippocampus sub-regions. Data are presented as mean ± SEM. Significance: *****p* ≤ 0.0001. CA1 ANOVA; *F*(3, 28) = 64.36, *p* < 0.0001, CA3 ANOVA; *F*(3, 19) = 21.09, *p* < 0.0001, Hilus ANOVA; *F*(3, 28) = 15.63, *p* < 0.0001, IBA1 total surface ANOVA, *F*(3, 67) = 14.90, *p* < 0.0001.

Supplementary Figure 3 The epifluorescence analysis of P-JNK and P-ERK. **(A)** Representative confocal microscopic images of the immunoreactivity of p-JNK (green) in scopolamine-treated mouse brains. Scale bar; 100 μm. **(B)** Analysis of the fluorescence intensity of p-JNK in the CA1, CA3 and hilus regions. Data are presented as mean ± SEM. **(C)** Representative confocal microscopic images of the immunoreactivity of p-ERK (green) in scopolamine-treated mouse brains. Scale bar; 100 μm. **(D)** Analysis of the fluorescence intensity of p-ERK in the CA1, CA3 and hilus regions. Data are presented as mean ± SEM.

## References

[B1] AmeliMojarad M. AmeliMojarad M. (2024). The neuroinflammatory role of microglia in Alzheimer’s disease and their associated therapeutic targets. *CNS Neurosci. Ther.* 30:e14856. 10.1111/cns.14856 39031970 PMC11259573

[B2] Balestrieri J. V. L. Nonato M. B. Gheler L. Prandini M. N. (2020). Structural volume of hippocampus and Alzheimer’s disease. *Rev. Assoc. Med. Bras.* 66 512–515. 10.1590/1806-9282.66.4.512 32578788

[B3] Carter S. F. Herholz K. Rosa-Neto P. Pellerin L. Nordberg A. Zimmer E. R. (2019). Astrocyte biomarkers in Alzheimer’s disease. *Trends Mol. Med.* 25 77–95. 10.1016/j.molmed.2018.11.006 30611668

[B4] Crawford T. J. Higham S. (2016). Distinguishing between impairments of working memory and inhibitory control in cases of early dementia. *Neuropsychologia* 81 61–67. 10.1016/j.neuropsychologia.2015.12.007 26687733

[B5] DeTaboada L. Ilic S. Leichliter-Martha S. Oron U. Oron A. Streeter J. (2006). Transcranial application of low-energy laser irradiation improves neurological deficits in rats following acute stroke. *Lasers Surg. Med.* 38 70–73. 10.1002/lsm.20256 16444697

[B6] Endo F. Kasai A. Soto J. S. Yu X. Qu Z. Khakh B. S. (2022). Molecular basis of astrocyte diversity and morphology across the CNS in health and disease. *Science* 378:eadc9020. 10.1126/science.adc9020 36378959 PMC9873482

[B7] Fellgiebel A. Yakushev I. (2011). Diffusion tensor imaging of the hippocampus in MCI and early Alzheimer’s disease. *J. Alzheimers Dis.* 26 257–262. 10.3233/jad-2011-0001 21971465

[B8] Flood J. F. Cherkin A. (1986). Scopolamine effects on memory retention in mice: A model of dementia? *Behav. Neural Biol.* 45 169–184. 10.1016/s0163-1047(86)90750-8 3964171

[B9] Francis P. T. Palmer A. M. Snape M. Wilcock G. K. (1999). The cholinergic hypothesis of Alzheimer’s disease: A review of progress. *J. Neurol. Neurosurg. Psychiatry* 66 137–147. 10.1136/jnnp.66.2.137 10071091 PMC1736202

[B10] Ghoneim M. M. Mewaldt S. P. (1977). Studies on human memory: The interactions of diazepam, scopolamine, and physostigmine. *Psychopharmacology* 52 1–6. 10.1007/BF00426592 403551

[B11] Hamblin M. R. (2016). Shining light on the head: Photobiomodulation for brain disorders. *BBA Clin.* 6 113–124. 10.1016/j.bbacli.2016.09.002 27752476 PMC5066074

[B12] Hong N. Kang G. W. Park J. O. Chung P. S. Lee M. Y. Ahn J. C. (2022). Photobiomodulation regulates adult neurogenesis in the hippocampus in a status epilepticus animal model. *Sci. Rep.* 12:15246. 10.1038/s41598-022-19607-5 36085308 PMC9463127

[B13] Hong N. Kim H. J. Kang K. Park J. O. Mun S. Ahn J. C. (2023). Photobiomodulation improves the synapses and cognitive function and ameliorates epileptic seizure by inhibiting downregulation of Nlgn3. *Cell Biosci.* 13:8. 10.1186/s13578-022-00949-6 36635704 PMC9837965

[B14] Hyman B. T. (1997). The neuropathological diagnosis of Alzheimer’s disease: Clinical-pathological studies. *Neurobiol. Aging* 4 S27–S32. 10.1016/s0197-4580(97)00066-3 9330982

[B15] Kempuraj D. Thangavel R. Natteru P. A. Selvakumar G. P. Saeed D. Zaheer A. (2016). Neuroinflammation induces neurodegeneration. *J. Neurol. Neurosurg. Spine* 1:1003. PMC5260818 28127589

[B16] Kim E. K. Choi E. J. (2010). Pathological roles of MAPK signaling pathways in human diseases. *Biochim. Biophys. Acta* 1802 396–405. 10.1016/j.bbadis.2009.12.009 20079433

[B17] Klinkenberg I. Blokland A. (2010). The validity of scopolamine as a pharmacological model for cognitive impairment: A review of animal behavioral studies. *Neurosci. Biobehav. Rev.* 34 1307–1350. 10.1016/j.neubiorev.2010.04.001 20398692

[B18] Liddelow S. A. Barres B. A. (2017). Reactive astrocytes: Production, function, and therapeutic potential. *Immunity* 46 957–967. 10.1016/j.immuni.2017.06.006 28636962

[B19] Luppi J. J. Schoonhoven D. N. van Nifterick A. M. Gouw A. A. Hillebrand A. de Haan W. (2022). Oscillatory activity of the hippocampus in prodromal Alzheimer’s disease: A source-space magnetoencephalography study. *J. Alzheimers Dis.* 87 317–333. 10.3233/jad-215464 35311705 PMC9198749

[B20] Mester E. Nagylucskay S. Doklen A. Tisza S. (1976). Laser stimulation of wound healing. *Acta Chir. Acad. Sci. Hung* 17 49–55. 970061

[B21] Murphy M. P. LeVine H. III (2010). Alzheimer’s disease and the amyloid-beta peptide. *J. Alzheimers Dis.* 19 311–323. 10.3233/JAD-2010-1221 20061647 PMC2813509

[B22] Nedergaard M. Verkhratsky A. (2012). Artifact versus reality–how astrocytes contribute to synaptic events. *Glia* 60 1013–1023. 10.1002/glia.22288 22228580 PMC3340515

[B23] Oberheim N. A. Tian G. F. Han X. Peng W. Takano T. Ransom B. (2008). Loss of astrocytic domain organization in the epileptic brain. *J. Neurosci.* 28 3264–3276. 10.1523/JNEUROSCI.4980-07.2008 18367594 PMC6670598

[B24] Perry V. H. Nicoll J. A. Holmes C. (2010). Microglia in neurodegenerative disease. *Nat. Rev. Neurol.* 6 193–201. 10.1038/nrneurol.2010.17 20234358

[B25] Pitsikas N. (2015). The role of nitric oxide in the object recognition memory. *Behav. Brain Res.* 285 200–207. 10.1016/j.bbr.2014.06.008 24933185

[B26] Raingeaud J. Gupta S. Rogers J. S. Dickens M. Han J. Ulevitch R. J. (1995). Pro-inflammatory cytokines and environmental stress cause p38 mitogen-activated protein kinase activation by dual phosphorylation on tyrosine and threonine. *J. Biol. Chem.* 270 7420–7426. 10.1074/jbc.270.13.7420 7535770

[B27] Rao Y. L. Ganaraja B. Murlimanju B. V. Joy T. Krishnamurthy A. Agrawal A. (2022). Hippocampus and its involvement in Alzheimer’s disease: A review. *3 Biotech* 12:55. 10.1007/s13205-022-03123-4 35116217 PMC8807768

[B28] Rountree S. D. Chan W. Pavlik V. N. Darby E. J. Siddiqui S. Doody R. S. (2009). Persistent treatment with cholinesterase inhibitors and/or memantine slows clinical progression of Alzheimer disease. *Alzheimers Res. Ther.* 1:7. 10.1186/alzrt7 19845950 PMC2874259

[B29] Saxena G. Singh S. P. Agrawal R. Nath C. (2008). Effect of donepezil and tacrine on oxidative stress in intracerebral streptozotocin-induced model of dementia in mice. *Eur. J. Pharmacol.* 581 283–289. 10.1016/j.ejphar.2007.12.009 18234183

[B30] Steele M. L. Robinson S. R. (2012). Reactive astrocytes give neurons less support: Implications for Alzheimer’s disease. *Neurobiol. Aging* 33:e00421–13. 10.1016/j.neurobiolaging.2010.09.018 21051108

[B31] Tang K. S. (2019). The cellular and molecular processes associated with scopolamine-induced memory deficit: A model of Alzheimer’s biomarkers. *Life Sci.* 233:116695. 10.1016/j.lfs.2019.116695 31351082

[B32] Waypa G. B. Smith K. A. Schumacker P. T. (2016). O2 sensing, mitochondria and ROS signaling: The fog is lifting. *Mol. Aspects Med.* 47-48 76–89. 10.1016/j.mam.2016.01.002 26776678 PMC4750107

[B33] Yoon S. R. Chang S. Y. Lee M. Y. Ahn J. C. (2023). Effects of 660-nm LED photobiomodulation on drebrin expression pattern and astrocyte migration. *Sci. Rep.* 13:6220. 10.1038/s41598-023-33469-5 37069238 PMC10110518

[B34] Yoon S. R. Hong N. Lee M. Y. Ahn J. C. (2021). Photobiomodulation with a 660-nanometer light-emitting diode promotes cell proliferation in astrocyte culture. *Cells* 10:1664. 10.3390/cells10071664 34359834 PMC8307591

[B35] Zhu X. Lee H. G. Raina A. K. Perry G. Smith M. A. (2002). The role of mitogen-activated protein kinase pathways in Alzheimer’s disease. *Neurosignals* 11 270–281. 10.1159/000067426 12566928

[B36] Zhuang Z. Y. Wen Y. R. Zhang D. R. Borsello T. Bonny C. Ji R. R. (2006). A peptide c-Jun N-terminal kinase (JNK) inhibitor blocks mechanical allodynia after spinal nerve ligation: Respective roles of JNK activation in primary sensory neurons and spinal astrocytes for neuropathic pain development and maintenance. *J. Neurosci.* 26 3551–3560. 10.1523/JNEUROSCI.5290-05.2006 16571763 PMC6673862

