# Supplementary material for: Photobiomodulation regulates astrocyte activity and ameliorates scopolamine-induced cognitive behavioral decline
Source: Front Cell Neurosci. 2024 Sep 20;18:1448005. doi: 10.3389/fncel.2024.1448005 (PMC11449862; doi:10.3389/fncel.2024.1448005)
Supplement: Supplementary file 1 [file Presentation_1.PDF]

Supplementary figure 1

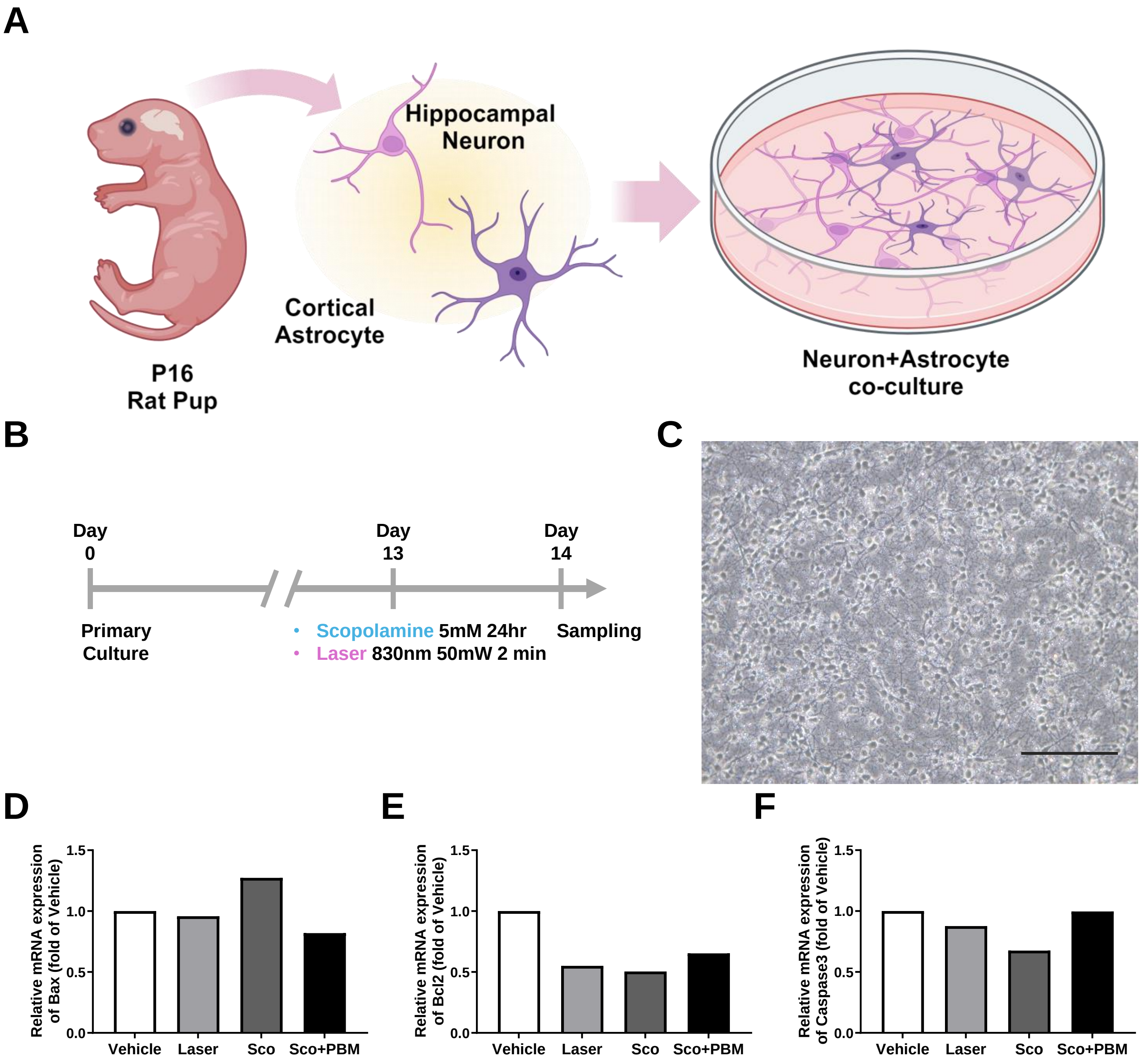

Supplementary figure 2

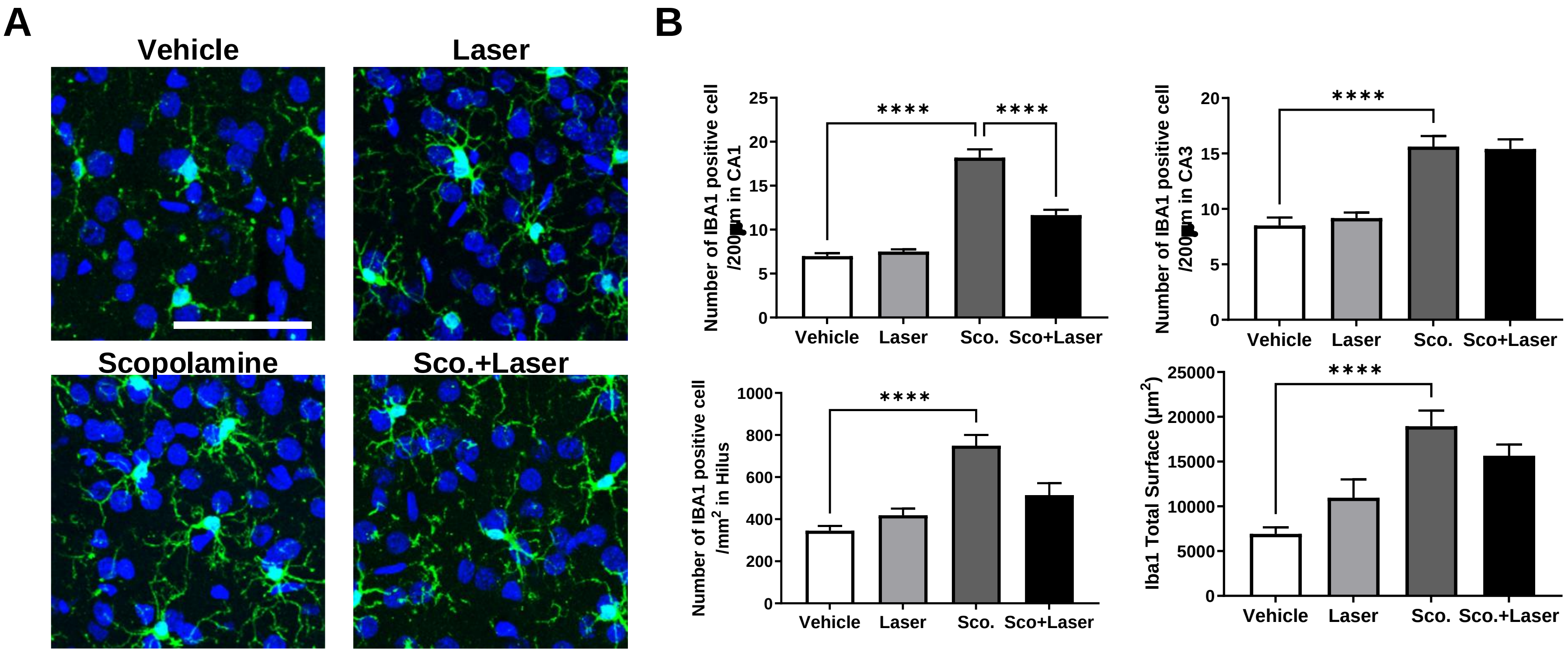

Supplementary figure 3

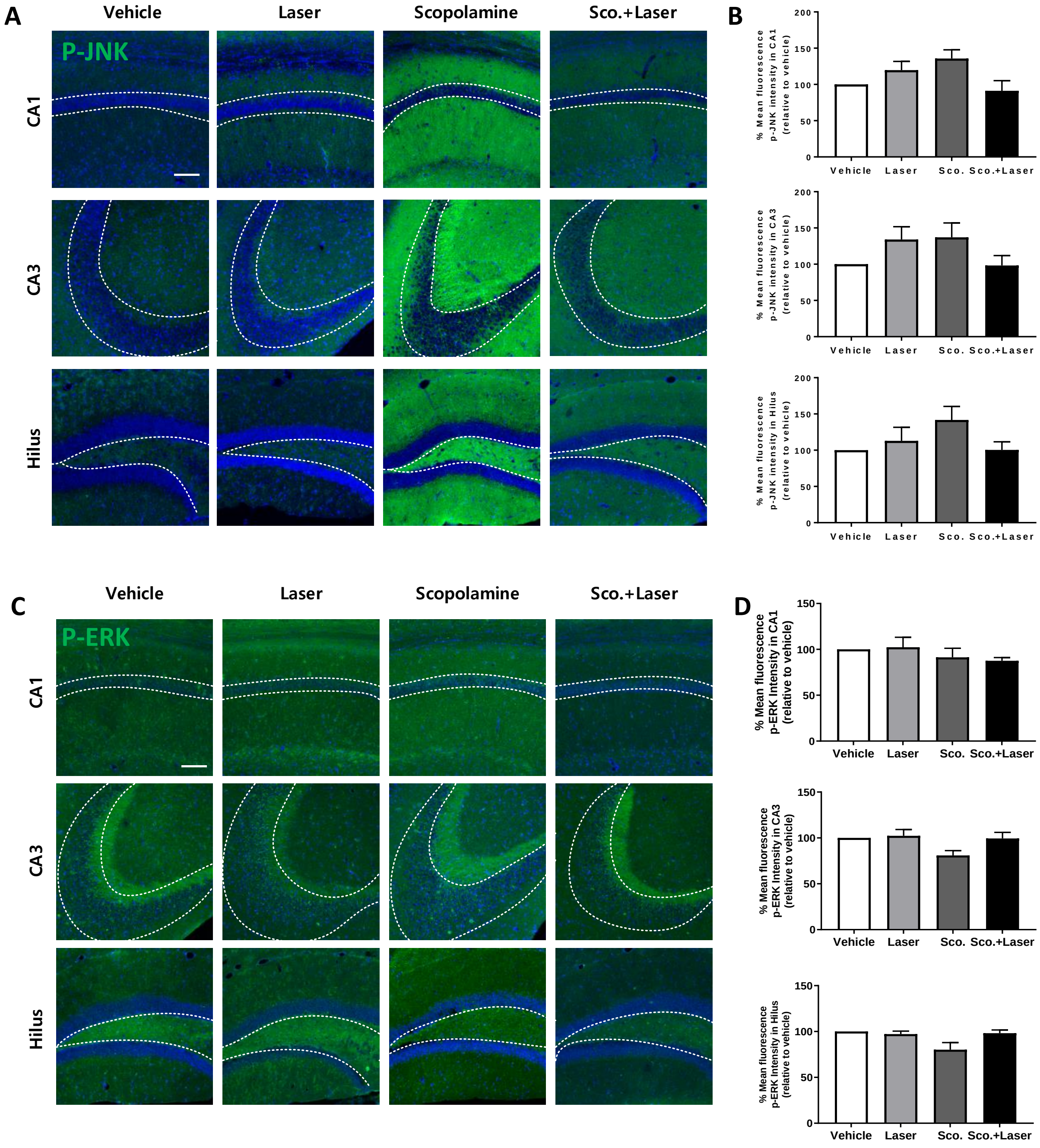

Supplementary Table 1. Primer sequence for the real-time polymerase chain reaction.

Gene	Forward Primers	Reverse Primers	product length(bp)	NCBI Reference Sequence
GAPDH	GCACAGTCAAGGCTGAGAATG	ATGGTGGTGAAGACGCCAGTA	142	NM_017008.4
Bax	AGGCGAATTGGCGATGAACTGG	CTAGCAAAGTAGAAAAGGGCAACC	164	NM_017059.2
Bcl2	GTGGATGACTGAGTACCT	CCAGGAGAAATCAAACAGAG	118	NM_016993.2
Caspase3	GAGACAGACAGTGGAACTGACGATG	GGCGCAAAGTGACTGGATGA	147	NM_012922.2
